# Editorial: The bodyguards to the rescue: understanding the molecular and immunological mechanisms involved in host-insect pathogenic microbe interactions

**DOI:** 10.3389/fcimb.2025.1646507

**Published:** 2025-07-04

**Authors:** Bamisope Steve Bamisile, Muhammad Hafeez, Mubasher Hussain, Chandra Kanta Dash, Junaid Ali Siddiqui

**Affiliations:** ^1^ Department of Entomology and Plant Pathology, Universidade Estadual do Norte Fluminense Darcy Ribeiro, Campos dos Goytacazes, Brazil; ^2^ Department of Entomology, South China Agricultural University, Guangzhou, China; ^3^ Department of Agriculture, Veterinary and Rangeland Sciences, University of Nevada, Reno, NV, United States; ^4^ Department of Pest Control Engineer and Technology, Guangdong Pest Control Technology Group, Guangzhou, China; ^5^ Department of Entomology, Sylhet Agricultural University, Sylhet, Bangladesh; ^6^ The School of Biological Sciences, Faculty of Science, The Hong Kong University, Hong Kong, Hong Kong SAR, China

**Keywords:** entomopathogenic fungi, immune response, insect pest control, defense mechanisms, social insects

The articles featured in this Research Topic explored various aspects of biological control, including the mechanisms involved in host-pathogen interactions, the virulence of entomopathogenic microbes (specifically fungi and bacteria) against insects, and the immune response to infection by bioagents. The featured studies reported innovative and sustainable strategies for controlling insect pests and disease vectors, drawing on a deep understanding of the physiology of insect pests and biological control agents.

The application of bioagents for the regulation of natural insect pest populations and the management of disease-causing pathogens is considered relatively safer and more environmentally sustainable (Souza et al., 2023). Nevertheless, their efficacy is significantly influenced by various ecological factors, with biotic factors in the shared environment being particularly prominent (Rosenheim et al., 1995). The immune response of the target host is a critical consideration while developing biological control agents for insect management. For example, certain insects can detect and evade entomopathogenic microbes by relying on their sense of smell. When infected with fungi, ants exhibit self-medication behavior, increase social interactions with their nestmates, and alter their behavior in response to infection (Wang et al., 2015; Qiu et al., 2016). Furthermore, cuticular lipids may influence the pathogenicity of microorganisms that infiltrate the cuticle of the insect host (Keyhani, 2018; Pedrini, 2018). To overcome these constraints, researchers and pest management professionals are working to develop innovative strategies to improve the overall performance of biological control agents. These approaches include enhancing the virulence and host specificity of bioagents, improving delivery methods, and manipulating insect physiology. The articles featured in this Research Topic are focused on this direction and provided compelling insights into the diverse molecular and immunological mechanisms that govern host-insect-pathogenic microbe interactions. From parasitic wasps to fungal endophytes, from plant-derived antifungals to gut-associated bacteria, these studies demonstrated the complexity of multi-trophic interactions and the strategic roles the entomopathogenic microbes can play.

For example, Chepkemoi et al. evaluated the compatibility of several entomopathogenic fungal isolates with *Telenomus remus*, a key egg parasitoid of *Spodoptera frugiperda*, for the management of fall armyworm. Their findings revealed that, depending on the dose and exposure method, certain *Metarhizium anisopliae* and *Beauveria bassiana* strains significantly affected the survival and parasitism rates of *T. remus*. Overall, the study indicated the potential combination of both *M. anisopliae* and *B. bassiana* with *T. remus* parasitoids for effective suppression of *S. frugiperda* populations. The results underscore the importance of understanding host immune responses and microbial virulence mechanisms when combining biological control agents.

In a related study, Paweer et al. demonstrated that tomato plants endophytically colonized by *Hypocrea lixii* and *Trichoderma asperellum* showed enhanced systemic resistance against *Trialeurodes vaporariorum*, leading to significantly reduced transmission and severity of tomato infectious chlorosis virus and tomato chlorosis virus. This study highlighted the immunological and molecular roles of fungal endophytes in modulating host-vector-pathogen interactions under natural conditions.

Sepulveda et al. investigated the antifungal potential of aqueous extracts derived from *Capsicum* species. The authors revealed that the extracts were rich in capsaicinoids and phenolic compounds and exhibited significant fungistatic effects against plant pathogens and food spoilage fungi. In addition, the compounds altered fungal morphology but, interestingly, showed no significant ecotoxicity in *Galleria mellonella*. These findings provide important insights into the biochemical mechanisms by which plant-derived compounds can modulate fungal pathogenicity, supporting a broader understanding of host-pathogen interactions and microbial suppression mechanisms relevant to host-insect-pathogenic microbe dynamics.

Another interesting original research in this Research Topic reveals critical molecular and immunological mechanisms in host-microbe interactions and highlights the potential of utilizing insect-associated microbes for biological control. In this article, Li et al. demonstrated that *Bacillus velezensis* isolated from adult housefly intestines disrupted the gut microbiota, suppressed immune responses, and altered gene expression in housefly larvae. In addition, the isolated *Bacillus* sp. significantly impaired the development of house fly larvae and mediated an increase in mortality. The bacterial association with the larva significantly affected the larval transcriptome, modulating the expression of genes involved in various biological pathways, which are essential for insect development and survival, including macronutrient metabolism and energy production.

The other contribution is a Brief Research Report by Schiefermeier-Mach et al. Although not insect-focused, the article provides a critical lens into host-microbe interactions by analyzing how alveolar epithelial cell geometry and extracellular matrix composition influence the uptake of *Aspergillus fumigatus* conidia. The study revealed that spatial cell arrangement and boundary conditions affect fungal internalization and intracellular processing, emphasizing the role of mechanical and immunological cues in host-pathogen interactions. By uncovering how mechanical and biochemical boundary conditions regulate fungal-host cell interactions at the molecular level, this study deepens our understanding of host-pathogen immunological dynamics. In addition, these findings have broader implications for understanding similar processes in insect-pathogenic fungal infections.

In summary, the studies presented here demonstrate the complex molecular and immunological mechanisms underlying host-insect-pathogenic microbe interactions. Together, they not only advance our scientific understanding of these complex dynamics but also pave the way for more targeted, ecologically sound biological control strategies in integrated pest management systems.
